# Supplementary material for: Using the attachment network Q-sort for profiling one’s attachment style with different attachment-figures
Source: PLoS One. 2020 Sep 3;15(9):e0237576. doi: 10.1371/journal.pone.0237576 (PMC7470453; doi:10.1371/journal.pone.0237576)
Supplement: S1 File — (DOCX) [file pone.0237576.s001.docx]

Supplementary Material

S1: ANQ items

S2: Confirmatory Factor Analyses

Supplementary table 1a: Results of confirmatory factor analysis protocol for the ANQ questionnaire for the mother as attachment figure in the London sample (n=340).

Supplementary table 1b: Results of confirmatory factor analysis protocol for the ANQ questionnaire for the father as attachment figure in the London sample (n=340).

Supplementary table 1c: Results of confirmatory factor analysis protocol for the ANQ questionnaire for the romantic partner as attachment figure in the London sample (n=340).

S3: Multi-group CFA Modelling, excluding romantic partner as attachment figure

Supplementary table 2a: Model Fit statistics of multiple group measurement and structural invariance for mother and father as attachment figure in the London sample.

Supplementary table 2b: Model Fit statistics of multiple group measurement and structural invariance for mother and father as attachment figure in the full sample.

Supplementary table 2c: Model Fit statistics of multiple group measurement and structural invariance of the ANQ for the London, Rotterdam patient and Rotterdam healthy control samples.

Supplementary table 2d: Model Fit statistics of multiple group measurement and structural invariance for mother, father, and romantic partner as attachment figure in the London sample.

Supplementary table 2e: Model Fit statistics of multiple group measurement and structural invariance for mother, father and romantic partner as attachment figure in the full sample.

Supplementary table 2f: Model Fit statistics of multiple group measurement and structural invariance of the ANQ for the London, Rotterdam patient and Rotterdam healthy control samples.

S4: Supplementarytable 3 ANQ-sort attachment style scale scores per attachment style profile group

S5: Scoring Tool ANQ (Excel file), not included in this document.

S1 APPENDIX: ANQ items

Items retained after confirmative factor analysis are represented in bold

SECURE

**ANQ1: When I need him/her he always makes time for me**

ANQ2: I am confident that our relationship will last

**ANQ3: I know he/she won't abandon me**

**ANQ4: I like being able to depend on him/her for emotional support**

ANQ5: When I am sick, I am comfortable depending on him/her

**ANQ6: I trust him/her completely**

**ANQ7: He/she never hurts my feelings when I turn to him/her for help**

**ANQ8: I know he/she won't let me down**

**ANQ9: I am confident that he/she will really understand my feelings**

**ANQ10: He/she pays attention to my needs**

**ANQ11: Whenever I tell him/her my personal problems. I know he/she is concerned**

ANQ12: I feel good knowing that he/she cares about me

**ANQ13: When I am frightened I feel safer with him/her**

**ANQ14:** **I find it easy to get emotionally close to him/her**

**ANQ15:** **I turn to him/her for comfort and reassurance**

**ANQ16:** **When I am sad, he/she comforts me**

**ANQ17:** **When I feel anxious, being close to him/her makes me feel better**

**ANQ18:** **When I am hurting, talking to him/her makes me feel better**

**ANQ19:** **He/she is the first person I look for when something bad happens**

ANQ20: When I am hurting I really want to tell him/her

DISMISSIVE

**ANQ21:** **I don't care whether or not I have his/her support**

ANQ22: It isn't Important to me whether he/she cares about me

**ANQ23:** **I wouldn’t care if our relationship ended**

**ANQ24:** **I don't need him/her to be there for me**

**ANQ25:** **I don't need him/her to stay with me**

**ANQ26:** **I don't tell him/her when I'm hurting**

**ANQ27:** **I don't turn to him/her for help**

**ANQ28:** **I prefer to keep some distance between us**

ANQ29: I don't give him/her the chance to let me down

**ANQ30:** **When I'm upset, I don't turn to him/her for comfort**

PREOCCUPIED

**ANQ31:** **When I tell him/her my troubles I feel like he/she doesn't really care**

**ANQ32:** **Even when I spend time with him/her it doesn't make me feel secure**

**ANQ33:** **When I show my feelings for him/her, I worry that he/she will not feel the same about me**

**ANQ34:** **I often wonder whether he/she really cares about me**

ANQ35: Even though I know I will feel worse, I keep going back to him/her for support

ANQ36: I get frustrated with him/her when he's/she's not there for me

ANQ37: I feel angry when he/she doesn't pay enough attention to my needs

**ANQ38:** **Sometimes I have to force him/her to show more commitment to me**

ANQ39: I often have to get angry to get his/her attention

ANQ40: I will do anything to prevent him/her from leaving me

Non-attachment items (not included in latent class analyses).

POSITIVE NON-ATTACHMENT

ANQ41: I like to hear about what he/she does

ANQ42: We have a lot in common

ANQ43: I enjoy joking with him/her

ANQ44: I have a lot of fun with him/her

ANQ45: We enjoy eating together

ANQ46: We work well together

ANQ47: I find him/her attractive

ANQ48: I'm a better person for knowing him/her

ANQ49: I admire his/her competence

ANQ50: He/she Is enormously helpful with problem solving

NEGATIVE NON-ATTACHMENT

ANQ51: He/she is not very interesting to be with

ANQ52: It's hard for us to cooperate

ANQ53: I don't like him/her

ANQ54: He/she Is extremely annoying to me

ANQ55: I don't like his/her sense of humor

ANQ56: Some of his/her habits bug me

ANQ57: He's/she's a bad influence on me

ANQ58: He/she often bosses me around

ANQ59: He/she doesn't take my opinions seriously

ANQ60: He/she makes fun of me

S2 Appendix: Confirmatory Factor Analyses

Supplementary table 1a: Results of confirmatory factor analysis protocol for the ANQ questionnaire for the mother as attachment figure in the London sample (n=340).

|  | Model A | Model B | Model C | Model D | Model E | Model F | Model G |
| --- | --- | --- | --- | --- | --- | --- | --- |
| Number and content of factors tested | Theoretical model consisting of  3 factors  Secure (items 1-20); Dismissive (items 21-30); Preoccupied (items 31-40) | Model A, excluding items:  ANQ20  ANQ22  ANQ36  ANQ37  ANQ40 | Model B, with  factors Secure and Preoccupied combined into 1 factor | Model B, with factors  Dismissive and Preoccupied combined into 1 factor | Model B, excluding items:  ANQ2  ANQ5  ANQ12  ANQ29  ANQ35  ANQ39 | Model B, excluding items:  ANQ12  ANQ29  ANQ35  ANQ39 | Model F, excluding item:  ANQ7 |
| Chi2 | 1769 | 1260 | 1331 | 1352 | 962 | 1060 | 2540 |
| df | 737 | 557 | 559 | 559 | 374 | 431 | 404 |
| p | <0.001 | <0.001 | <0.001 | <0.001 | <0.001 | <0.001 | <0.001 |
| Chi2/df ratio | 2.400 | 2.262 | 2.381 | 2.419 | 2.572 | 2.459 | 6.287 |
| RMSEA (95%CI) | 0.064  (0.060 – 0.068) | 0.061 (0.056-0.065) | 0.064  (0.059- 0.068) | 0.065  (0.060-0.069) | 0.068 (0.063-0.073) | 0.066 (0.061-0.071) | 0.125 (0.120-0.129) |
| CFI | 0.878 | 0.915 | 0.907 | 0.904 | 0.921 | 0.919 | 0.716 |
| TLI | 0.871 | 0.909 | 0.901 | 0.898 | 0.914 | 0.912 | 0.694 |
| SRMR | 0.068 | 0.060 | 0.062 | 0.063 | 0.059 | 0.059 | 0.104 |
| Items with an R2 <.130 | ANQ20  ANQ22  ANQ36  ANQ37  ANQ40 | None | ANQ35 | ANQ35 | None | None | None |
| Items with an R2 <.200 | ANQ2  ANQ5  ANQ7  ANQ12  ANQ25  ANQ29  ANQ33  ANQ35  ANQ39 | ANQ2  ANQ5  ANQ7  ANQ12  ANQ25  ANQ29  ANQ33  ANQ35  ANQ39 | ANQ2  ANQ5  ANQ7  ANQ12  ANQ25  ANQ29  ANQ33  ANQ38  ANQ39 | ANQ2  ANQ5  ANQ7  ANQ12  ANQ25  ANQ29  ANQ33  ANQ38  ANQ39 | ANQ7 | ANQ2  ANQ5  ANQ7  ANQ25  ANQ33  ANQ38 | ANQ2  ANQ5  ANQ25  ANQ33  ANQ38 |
| Comments |  |  | Modification Indices indicate no improvement for items of combined factor | Modification Indices indicate no improvement for items of combined factor |  | Minimal set of excluded items, e.g. only those items that generated R2 <0.200 for mother, father and partner |  |

Supplementary table 1b: Results of confirmatory factor analysis protocol for the ANQ questionnaire for the father as attachment figure in the London sample (n=340).

|  | Model A | Model B | Model C | Model D | Model E | Model F | Model G |
| --- | --- | --- | --- | --- | --- | --- | --- |
| Number and content of factors tested | Theoretical model consisting of  3 factors  Secure (items 1-20); Dismissive (items 21-30); Preoccupied (items 31-40) | Model A, excluding items:  ANQ20  ANQ22  ANQ36  ANQ37  ANQ40 | Model B, with  factors Secure and Preoccupied combined into 1 factor | Model B, with factors  Dismissive and Preoccupied combined into 1 factor | Model B, excluding items:  ANQ2  ANQ5  ANQ12  ANQ29  ANQ35  ANQ39 | Model B, excluding items:  ANQ12  ANQ29  ANQ35  ANQ39 | Model F, excluding item:  ANQ7 |
| Chi2 | 1629 | 1123 | 1160 | 1182 | 817 | 901 | 2653 |
| df | 737 | 557 | 559 | 559 | 374 | 431 | 404 |
| p | <0.001 | <0.001 | <0.001 | <0.001 | <0.001 | <0.001 | <0.001 |
| Chi2/df ratio | 2.210 | 2.016 | 2.075 | 2.114 | 2.184 | 2.090 | 6.567 |
| RMSEA | 0.060  (0.056 – 0.064) | 0.055 (0.050-0.059) | 0.056 (0.052-0.061) | 0.057 (0.053-0.062 | 0.059 (0.054-0.065) | 0.057 (0.051-0.062) | 0.128 (0.123-0.133) |
| CFI | 0.912 | 0.943 | 0.939 | 0.937 | 0.949 | 0.948 | 0.748 |
| TLI | 0.906 | 0.938 | 0.935 | 0.932 | 0.944 | 0.944 | 0.729 |
| SRMR | 0.063 | 0.055 | 0.056 | 0.057 | 0.052 | 0.052 | 0.103 |
| Items with an R2 <.130 | ANQ20  ANQ22  ANQ36  ANQ37  ANQ40 | None | None | ANQ29 | None | None | None |
| Items with an R2 <.200 | ANQ7  ANQ12  ANQ29 | ANQ7  ANQ12  ANQ29  ANQ39 | ANQ7  ANQ12  ANQ29  ANQ35  ANQ39 | ANQ7  ANQ12  ANQ21  ANQ35  ANQ39 | ANQ7 | ANQ7 | None |
| Comments |  |  | Modification Indices indicate no improvement for items of combined factor | Modification Indices indicate no improvement for items of combined factor |  | Minimal set of excluded items, e.g. only those items that generated R2 <0.200 for mother, father and partner |  |

Supplementary table 1c: Results of confirmatory factor analysis protocol for the ANQ questionnaire for the romantic partner as attachment figure in the London sample (n=340).

|  | Model A | Model B | Model C | Model D | Model E | Model F | Model G |
| --- | --- | --- | --- | --- | --- | --- | --- |
| Number and content of factors tested | model consisting of  3 factors  Secure (items 1-20); Dismissive (items 21-30); Preoccupied (items 31-40 | Model A, excluding items:  ANQ20  ANQ22  ANQ36  ANQ37  ANQ40 | Model B, with  factors Secure and Preoccupied combined into 1 factor | Model B, with factors  Dismissive and Preoccupied combined into 1 factor | Model B, excluding items:  ANQ2  ANQ5  ANQ12  ANQ29  ANQ35  ANQ39 | Model B, excluding items:  ANQ12  ANQ29  ANQ35  ANQ39 | Model F, excluding item:  ANQ4 |
| Chi2 | 1940 | 1397 | 1540 | 1693 | 1021 | 1154 | 977 |
| df | 737 | 557 | 559 | 559 | 374 | 431 | 347 |
| p | <0.001 | <0.001 | <0.001 | <0.001 | <0.001 | <0.001 | <0.001 |
| Chi2/df ratio | 2.632 | 2.508 | 2.755 | 3.029 | 2.730 | 2.677 | 2.816 |
| RMSEA | 0.069  (0.066 – 0.073) | 0.067 (0.062-0.071) | 0.072 (0.068-0.076) | 0.077 (0.073-0.081) | 0.071 (0.066-0.077) | 0.070 (0.065-0.075) | 0.073 (0.068-0.078) |
| CFI | 0.754 | 0.815 | 0.784 | 0.750 | 0.828 | 0.828 | 0.830 |
| TLI | 0.739 | 0.802 | 0.770 | 0.734 | 0.813 | 0.814 | 0.815 |
| SRMR | 0.079 | 0.071 | 0.074 | 0.078 | 0.069 | 0.069 | 0.069 |
| Items with an R2 <.130 | ANQ4  ANQ5  ANQ12  ANQ13  ANQ20  ANQ22  ANQ29  ANQ40 | ANQ4  ANQ5  ANQ12  ANQ13  ANQ29 | ANQ4  ANQ5  ANQ12  ANQ13  ANQ29  ANQ33 | ANQ4  ANQ5  ANQ12  ANQ13  ANQ18  ANQ21  ANQ24  ANQ26  ANQ28  ANQ29  ANQ33  ANQ38  ANQ39 | ANQ4  ANQ13 | ANQ4  ANQ5  ANQ13  ANQ18  ANQ33 | ANQ13  ANQ18  ANQ33 |
| Items with an R2 <.200 | ANQ1  ANQ7  ANQ14  ANQ16  ANQ19  ANQ33  ANQ35  ANQ36  ANQ37  ANQ38 | ANQ1  ANQ7  ANQ14  ANQ16  ANQ19  ANQ33  ANQ38 | ANQ1  ANQ1  ANQ7  ANQ14  ANQ18  ANQ19  ANQ21  ANQ35  ANQ38  ANQ39 | ANQ1  ANQ7  ANQ14  ANQ16  ANQ19  ANQ25  ANQ30  ANQ35 | ANQ1  ANQ7  ANQ14  ANQ18  ANQ21  ANQ33  ANQ38 | ANQ1  ANQ7  ANQ14  ANQ16  ANQ19  ANQ21  ANQ33  ANQ38 | ANQ1  ANQ7  ANQ14  ANQ19  ANQ21  ANQ38 |
| Comments |  |  | Modification Indices indicate no improvement for items of combined factor | Modification Indices indicate no improvement for items of combined factor |  | Minimal set of excluded items, e.g. only those items that generated R2 <0.200 for mother, father and partner |  |

Interpretation:

In balancing the statistical goodness of fit measures, the parsimonious assumption and the theoretical assumption, Model E, showed the best fit, with regards to mother and father as attachment figures. Acceptable fit was found regarding Chi2/df-ratio, RMSEA, CFI and SRMR. Fit according TLI was less optimal. This model fitted the theoretical factor structure excluding 11 ill-fitting items. Overall, the statistical fit of the models regarding the romantic partner was less optimal. That being said, Model E fitted the data with romantic partner as attachment figure best, with acceptable fit measures found regarding Chi2/df-ratio, RMSEA, and SRMR.

We found a strong negative relationship between the Secure attachment factor and the Preoccupied and Dismissive factors. In line with these findings, we saw a strong positive relationship between the factor dismissive and Preoccupied. However, combining the factors into a single factor did not result in better fit of the model (Model C).

S3 Appendix: Multi-group CFA Modelling, excluding romantic partner as attachment figure

Supplementary table 2a: Model Fit statistics of multiple group measurement and structural invariance for mother and father as attachment figure in the London sample. Attachment to mother was used as reference group.

|  | Configural invariance | Metric invariance:  Factor loading held equal | Scalar invariance:  Factor loading & intercepts held equal |
| --- | --- | --- | --- |
| Chi2 | 1668 | 1693 | 1863 |
| df | 748 | 774 | 803 |
| p-value | <0.001 | <0.001 | <0.001 |
| Scaling correction factor | 1.047 | 1.045 | 1.044 |
| RMSEA (95%CI) | 0.064 (0.059-0.069) | 0.066 (0.062-0.070) | 0.065 (0.060-0.070) |
| CFI | 0.935 | 0.934 | 0.913 |
| TLI | 0.929 | 0.927 | 0.903 |
| SRMR | 0.055 | 0.057 | 0.065 |
| ΔChi2 (SB-adjusted)* |  | 23 | 198 |
| Δdf |  | 26 | 55 |
| p-value |  | 0.63 | <0.001 |
| ΔCFI |  | 0.001 | 0.022 |
| ΔRMSEA |  | 0.002 | 0.001 |

* SB: Satorra-Bentler adjustment

Supplementary table 2b: Model Fit statistics of multiple group measurement and structural invariance for mother and father as attachment figure in the full sample. Attachment to mother was used as reference group.

|  | Configural invariance | | Metric invariance:  Factor loading held equal | Scalar invariance:  Factor loading & intercepts held equal |
| --- | --- | --- | --- | --- |
| Chi2 | 2067 | | 2099 | 2322 |
| df | 748 | | 774 | 803 |
| p-value | <0.001 | | <0.001 | <0.001 |
| Scaling correction factor | 1.048 | | 1.048 | 1.047 |
| RMSEA (95%CI) | 0.062 (0.058-0.066) | | 0.063 (0.059-0.067) | 0.072 (0.068-0.076) |
| CFI | 0.934 | | 0.930 | 0.828 |
| TLI | 0.928 | | 0.923 | 0.829 |
| SRMR | 0.051 | | 0.067 | 0.078 |
| ΔChi2 (SB-adjusted) |  | | 32 | 256 |
| Δdf |  | | 26 | 55 |
| p-value |  | | 0.193 | <0.001 |
| ΔCFI |  | 0.005 | | 0.106 |
| ΔRMSEA |  | 0.001 | | 0.010 |

* SB: Satorra-Bentler adjustment

Supplementary table 2c: Model Fit statistics of multiple group measurement and structural invariance of the ANQ for the London, Rotterdam patient and Rotterdam healthy control samples. The London sample was used as reference group. Only mother and father as attachment figures used.

|  | Configural invariance | Metric invariance:  Factor loading held equal | Scalar invariance:  Factor loading & intercepts held equal |
| --- | --- | --- | --- |
| Chi2 | 2571 | 2652 | 3118 |
| df | 1122 | 1174 | 1232 |
| p-value | <0.001 | <0.001 | <0.001 |
| Scaling correction factor | 1.031 | 1.033 | 1.033 |
| RMSEA (95%CI) | 0.061 (0.056-0.066) | 0.060 (0.056-0.064) | 0.065 (0.060-0.070) |
| CFI | 0.932 | 0.930 | 0.907 |
| TLI | 0.926 | 0.927 | 0.911 |
| SRMR | 0.061 | 0.059 | 0.064 |
| ΔChi2 (SB-adjusted) |  | 83 | 1053 |
| Δdf |  | 52 | 110 |
| p-value |  | 0.004 | <0.001 |
| ΔCFI |  | 0.002 | 0.025 |
| ΔRMSEA |  | 0.002 | 0.003 |

* SB: Satorra-Bentler adjustment

Supplementary table 2d: Model Fit statistics of multiple group measurement and structural invariance for mother, father, and romantic partner as attachment figure in the London sample. Attachment to mother was used as reference group.

|  | Configural invariance | Metric invariance:  Factor loading held equal | Scalar invariance:  Factor loading & intercepts held equal |
| --- | --- | --- | --- |
| Chi2 | 2539 | 2625 | 3495 |
| df | 1122 | 1174 | 1232 |
| p-value | <0.001 | <0.001 | <0.001 |
| Scaling correction factor | 1.043 | 1.047 | 1.046 |
| RMSEA (95%CI) | 0.061 (0.058-0.064) | 0.060 (0.057-0.063) | 0.074 (0.071-0.076) |
| CFI | 0.826 | 0.822 | 0.722 |
| TLI | 0.811 | 0.815 | 0.725 |
| SRMR | 0.065 | 0.073 | 0.096 |
| ΔChi2 (SB-adjusted)* |  | 88 | 936 |
| Δdf |  | 52 | 110 |
| p-value |  | 0.001 | <0.001 |
| ΔCFI |  | 0.004 | 0.104 |
| ΔRMSEA |  | 0.001 | 0.013 |

* SB: Satorra-Bentler adjustment

Supplementary table 2e: Model Fit statistics of multiple group measurement and structural invariance for mother, father and romantic partner as attachment figure in the full sample. Attachment to mother was used as reference group.

|  | Configural invariance | | Metric invariance:  Factor loading held equal | Scalar invariance:  Factor loading & intercepts held equal |
| --- | --- | --- | --- | --- |
| Chi2 | 3007 | | 3113 | 4363 |
| df | 1122 | | 1174 | 1232 |
| p-value | <0.001 | | <0.001 | <0.001 |
| Scaling correction factor | 1.046 | | 1.049 | 1.048 |
| RMSEA (95%CI) | 0.059 (0.057-0.062) | | 0.059 (0.056-0.061) | 0.073 (0.070-0.075) |
| CFI | 0.833 | | 0.828 | 0.722 |
| TLI | 0.818 | | 0.821 | 0.725 |
| SRMR | 0.065 | | 0.067 | 0.095 |
| ΔChi2 (SB-adjusted) |  | | 108 | 1336 |
| Δdf |  | | 52 | 110 |
| p-value |  | | <0.001 | <0.001 |
| ΔCFI |  | 0.005 | | 0.111 |
| ΔRMSEA |  | 0.000 | | 0.014 |

* SB: Satorra-Bentler adjustment

Supplementary table 2f: Model Fit statistics of multiple group measurement and structural invariance of the ANQ for the London, Rotterdam patient and Rotterdam healthy control samples. The London sample was used as reference group.

|  | Configural invariance | Metric invariance:  Factor loading held equal | Scalar invariance:  Factor loading & intercepts held equal |
| --- | --- | --- | --- |
| Chi2 | 3532 | 3631 | 4216 |
| df | 1122 | 1174 | 1232 |
| p-value | <0.001 | <0.001 | <0.001 |
| Scaling correction factor | 1.037 | 1.041 | 1.041 |
| RMSEA (95%CI) | 0.067 (0.064-0.069) | 0.066 (0.063-0.068) | 0.071 (0.069-0.073) |
| CFI | 0.804 | 0.801 | 0.758 |
| TLI | 0.788 | 0.793 | 0.761 |
| SRMR | 0.067 | 0.073 | 0.087 |
| ΔChi2 (SB-adjusted) |  | 103 | 671 |
| Δdf |  | 52 | 110 |
| p-value |  | <0.001 | <0.001 |
| ΔCFI |  | 0.003 | 0.118 |
| ΔRMSEA |  | 0.001 | 0.006 |

* SB: Satorra-Bentler adjustment

| **Supplementary Table 3 ANQ-sort attachment style scale scores per attachment style profile group** | | | | | |
| --- | --- | --- | --- | --- | --- |
|  | Overall Secure (OS)  N=263  (mean; SD) | Insecure for father (IF)  N=124  (mean; SD) | Insecure for mother (IM)  N=80  (mean; SD) | Insecure for father and mother (IFM)  N=43  (mean; SD) | statistic |
| secure M | 4.90 (.33) | 4.66 (.42) | 3.49 (.46) | 3.23 (.40) | F(3)=448.19; p<0.001 |
| dismissive M | 3.16 (.44) | 3.21 (.55) | 4.25 (.76) | 4.76 (.72) | F(3)=165.17; p<0.001 |
| preoccupied M | 2.80 (.48) | 3.23 (.67) | 4.40 (.69) | 4.53 (.61) | F(3)=224.70; p<0.001 |
| secure F | 4.68 (.39) | 3.30 (.47) | 4.53 (.46) | 3.16 (.40) | F(3)=401.42; p<0.001 |
| dismissive F | 3.28 (.52) | 4.56 (.70) | 3.44 (.60) | 4.78 (.80) | F(3)=174.47; p<0.001 |
| preoccupied F | 2.30 (.55) | 4.80 (.62) | 3.19 (.60) | 4.67 (.58) | F(3)=309.52; p<0.001 |
| secure partner | 4.69 (.56) | 4.61 (.49) | 4.51 (.64) | 4.43 (.69) | F(3)=3.25; p=0.022 |
| dismissive partner | 3.03 (.62) | 3.09 (.63) | 3.24 (.82) | 3.06 (.69) | F(3)=1.63; p=0.182 |
| preoccupied partner | 3.25 (.77) | 3.35 (.64) | 3.36 (.84) | 3.56 (.92) | F(3)=2.016; p=0.111 |
