# Supplementary material for: Using the attachment network Q-sort for profiling one’s attachment style with different attachment-figures
Source: PLoS One. 2020 Sep 3;15(9):e0237576. doi: 10.1371/journal.pone.0237576 (PMC7470453; doi:10.1371/journal.pone.0237576)
Supplement: Attachment — Submitted filename: response to reviewers.docx [file pone.0237576.s003.docx]

Alexandra Kafushansky, PhD

Academic Editor

PLOS ONE

Rebuttal Letter

Subject: manuscript “Using the Attachment Network Q-sort for profiling one’s attachment style with different attachment-figures’ (PONE-D-19-35791)

Dear Dr Kafushansky,

First of all, we would like to thank you for the careful attention that has been paid to our manuscript and we apologize that the first submission, by mistake, did not meet the style requirements of the journal.

In the next paragraphs we indicate how we made use of the comments of the editor and the reviewer to improve the manuscript.

Editor #1

We checked the PLOS ONE’s style requirements and prepared the manuscript accordingly.

Editor #2

The ANQ items are included in Appendix S1. As these are not under copyright, we preferred to leave Appendix S1 as it is for clarity and readability of the manuscript.

Editor #3

Accidently, initially the manuscript was submitted in landscape format. The layout is now corrected in portrait format.

Editor #4

Unfortunately, ethical restrictions and patient confidentiality prohibit us from making the databases publicly accessible. All data are stored at the institutional database of the Erasmus Medical Centre, and will be made available upon request. At the end of the methods section we inform the interested reader how to get access to the files. Additionally, we have provided a maximum of information in our supplementary material.

Editor additional comments:

We re-drafted the Discussion to make it more congruent with the hypothesis part of the Introduction.

- In the Abstract we added that the study is done in psychotherapy patients as well as in non-patient respondents.
- In the Methods section and in the Discussion, we explained that the study was done in two samples (an English convenient sample from the general population and a Dutch sample of psychotherapy patients and of healthy respondents) taken together to ensure generisability of the instrument and the structures yielded by the study.
- In the Methods section we explicated that in the Dutch sample DSM-IV criteria were applied necessary for the in- and exclusion criteria in the larger study of which this psychometric study was a part.
- The statistics paragraph is checked for completeness. The tests the editor is referring to are described as follows: ‘Association between the obtained attachment subgroups and proximal variables were formally tested using Chi2-tests for categorical variables, ANOVA’s for normally distributed continuous variables, and Kruskal-Wallis tests for non-normally distributed continuous data. ’

Reviewer Introduction bullet 1 and 2

The reviewer is completely right in assuming that the most important quality of the ANQ is its focus on assessing relationship-specific attachment styles. We now emphasize this more strongly in the Discussion.

Reviewer Introduction bullet 3.

In the construction of the ANQ the distinction between items that theoretically were considered attachment items versus non-attachment items, was assumed to be important. However, this distinction is empirically not confirmed as both sets of items are highly correlated with each other. As shown in table 3, and accompanying text in the Results section discriminant validity of the ANQ results regarding the non-attachment items was not shown This is now more clearly explained in the Discussion.

Reviewer Introduction bullet 4

A description of the scoring of the ANQ is added in the methods section.

Reviewer Method bullet 1

We now explain throughout the manuscript that the ANQ assesses current attachment styles towards the key-figures chosen.

Reviewer Method bullet 2

In the Discussion we added our reasons why the more elaborate ANQ could be an alternative for the ECR(-r) which indeed is shorter.

Reviewer Results bullet 1

In the Results section we removed a detailed description of the results on the DAPPsf to improve the readability of the manuscript. The details themselves are still presented in the table.

Reviewer Results bullet 2

The reviewer is correct. Preoccupied and dismissive subscale scores point generally in the same direction. However, the confirmative factor analysis showed that combining the dismissive and preoccupied subscales into a single insecure subscale did not result in a better fit (Model C in S2 tables 1a-c, see also model interpretation at the end of S2). This statistical argument, in combination with the underlying theory of ANQ which distinguished a preoccupied and dismissive component, resulted in our decision to retain both subscales in the latent class analyses. It might be possible that a larger dataset might have resulted in more classes, both in terms of differentiation between the preoccupied and dismissive subscale, as well as differentiation between attachment figures.

Reviewer Results bullet 3

We understand the comment of the reviewer on the detailed information given. We tried to shorten the text taking this into consideration in the Results and Discussion sections of the manuscript but to retain the analytic information in the tables. If the editor prefers, we are willing to remove this table from the text and move it to the appendix.

Reviewer Discussion bullet 1

We understand that the discussion of the supposed importance of attachment versus non-attachment items could be much better. We tried to improve this in the Results and Discussion sections.

Reviewer Discussion bullet 2

We added in the Discussion possible applications of the ANQ.

We thank you for the comments and suggestions to improve the manuscript and we tried to use them as good as possible. We send you a revised manuscript with track changes as well as ‘a clean’ revised manuscript.

Yours sincerely, and on behalf of my colleagues,

CG Kooiman
